# Psychedelic integration: An analysis of the concept and its practice

**DOI:** 10.3389/fpsyg.2022.824077

**Published:** 2022-08-04

**Authors:** Geoff J. Bathje, Eric Majeski, Mesphina Kudowor

**Affiliations:** ^1^Department of Counseling and Integrated Programs, Adler University, Chicago, IL, United States; ^2^Sana Healing Collective, Chicago, IL, United States

**Keywords:** psychedelic, integration, integrate, psychedelic assisted therapy, entheogen

## Abstract

The concept of integration has garnered increased attention in the past few years, despite a long history of only brief mention. Integration services are offered by therapists, coaches, and other practitioners, or may be self-guided. There are many definitions of psychedelic integration, and the term encompasses a range of practices and techniques. This seems to have led to confusion about what integration is and how it is best practiced. The primary focus of this manuscript is the presentation of the first extensive review and concept analysis of definitions, practices, and models of psychedelic integration. We provide a synthesized definition of integration, synthesized model of integration, and comprehensive summary of integration practices to bring clarity to the subject.

## Introduction

Psychedelic substances are garnering increased interest in Western societies after decades of suppression under the War on Drugs. Research and wide cultural use of psychedelics peaked in the 1960’s. This era produced more than 1,000 clinical papers covering 40,000 patients and six international conferences on psychedelic drug therapy (Grinspoon and Bakalar, 1979). The impact on art and culture is more difficult to quantify, though in the late 1970s about 10% of high school seniors reported they had used LSD (Nuwer, 2020). Research and broad cultural interest appear to be peaking again in the 2010s and 2020s. A number of psychedelic substances, including psilocybin, ketamine, MDMA, ayahuasca, and DMT, have completed or are undergoing clinical trials for the treatment of mental disorders, with meta-analysis revealing “large” to “very large” effect sizes (Luoma et al., 2020). A substantial curtailing of drug war policies is underway as a number of cities have passed “Decriminalize Nature” ordinances to make the enforcement of plant-based psychedelics the lowest law enforcement priority, and as Oregon, California, and several other states pursue state-wide decriminalization and legalization approaches (Aday et al., 2020). With increasing interest in psychedelics, particularly for psychotherapeutic purposes, and the corresponding expansion of clinics offering ketamine administration or take-home prescription that often include little or no preparation, psychological support, or integration (Ryan and Bennett, 2020), the topic of psychedelic integration is becoming ever more important.

The term “integration” has been utilized to describe the period following a psychedelic experience. A wide range of definitions has been put forward, characterized by an even wider variety of integrative practices, historically without models or guidance as to when and why particular practices might be chosen. Integration is often briefly mentioned in books and articles compared to preparing for, navigating, or facilitating psychedelic experiences, despite being widely cited as very important to retaining benefits and working through psychedelic experiences. In a recent survey of clinicians who received training in psychedelic-assisted therapies, the majority stated a desire for a better understanding of the concept of integration (Ali et al., 2021).

Modern research on the psychotherapeutic use of psychedelics attests to the value of an integration focus. Each of the longstanding organizations funding or organizing psychedelic research have developed treatment protocols that provide time for preparation, psychological support/psychotherapy, and integration, including The Multidisciplinary Association for Psychedelic Studies, the Beckley Foundation, and Heffter Research Institute (Coder, 2017). This focus on the therapeutic process, including integration, was influenced by the work of earlier practitioners and researchers who began developing psychotherapeutic approaches to psychedelics in the 1960s, such as Grof (1980), Metzner (2015), and Richards (2015). While therapists appear particularly interested in integration, we will address the limitations of traditional psychotherapy models as an exclusive basis for integration.

### Indigenous cultures

Indigenous use of psychedelics is not a phenomenon of the past, with a number of cultures having longstanding use up to the present day. For example, evidence of the earliest use of peyote by Native Americans, which the Native American Church still utilizes, dates back 5,700–10,000 years (Bruhn et al., 2002). Psychedelic substances have been used by Indigenous cultures for a variety of purposes, including sorcery, spiritual purposes, resolving physical ailments, building alliances, or engaging with culture (Labate and Cavnar, 2014). Broadly, Indigenous healing practices are said to typically rely on three core assumptions (holism, interconnectedness, and harmony/balance) and three primary approaches (heavy reliance on communal, group, and family networks, use of spiritual belief and tradition, and the inclusion of shamans; Sue et al., 2019). Furthermore, mind, body, community, spirit, and nature tend to be seen as a unified entity, with daily life, spirituality, and medicine also not seen as separate. Imbalance or separation between these elements is often seen as a source of illness (Sue et al., 2019).

With regard to integration, it is sometimes said that Indigenous peoples do not have the same need for formal integration because psychedelics are utilized within the community and with a more holistic worldview or because psychedelics are already more integrated into Indigenous cultures that utilize them (Aixala, 2017). In contrast to Indigenous cultures that have a tradition with psychedelics, Western participants may not possess adequate cultural references to comprehend complex, abstract, and symbolic content that often emerges with psychedelics, necessitating therapeutic support throughout the process, including during the integration stage (Loizaga-Velder and Pazzi, 2014). Still, we must acknowledge that treating integration as a separate phase of psychedelic experiences probably imposes Western dualistic thinking. In order to address imbalance and support the realignment of self, shamanic cultures may employ symbolism and rituals already entrenched in the culture, such as hypnosis or trance-like states, drumming, chanting, spirit manifestations, among others, all within a communal context, which stimulate engagement with the innate drive toward physiological, spiritual and social experiences (Winkelman, 2021). These practices may take place before, during, or after experience with the actual psychedelic substance. Contrary to occasional suggestions that integration is only relevant to Westerners, this would seem to suggest that Indigenous cultures tend to be rooted in philosophies and worldviews that encourage an ongoing orientation toward balance and integration. Balance, here, refers to the interconnectedness and harmonious interaction between the physical, emotional, relational, spiritual, and mental aspects of the human experience (Settee and Shukla, 2020). This perspective could be of great value to the more dualistic and linear thinking in Western cultures (Limb and Hodge, 2008).

Nevertheless, Western interest in Indigenous ceremonies, such as ayahuasca shamanism in Peru, has raised concerns about the impact of psychedelic tourism. The unequal, disruptive, and extractive nature of capitalist consumerism has led to reports of negative economic, social, cultural, health, and environmental impact on Indigenous communities from the presence of culturally and linguistically uninformed tourists and their disproportionate wealth (Gurvicius, 2015). As a result, there has been a recent focus on justice and reciprocity regarding Indigenous knowledge, as opposed to extractive approaches that either neglect or harm Indigenous peoples (Martinez, 2021).

### Western cultures

Recent research on the psychotherapeutic use of psychedelics has garnered much positive press in several Western cultures. It seems likely that this has shaped expectations that psychedelics are primarily useful for psychotherapeutic purposes. It is also possible that the existing understanding of pharmaceutical drugs has impacted expectations of how psychedelics work (e.g., as working via direct biomedical effects on passive patients). This idea may run contrary to more complex models of substance, set, and setting, where preparatory processes or intentions are thought to significantly influence psychedelic effects (Hartogsohn, 2017), and may not promote adequate expectations for the value of a facilitator or guide, or active engagement in an integration process afterward. Beyond this therapeutic interest, there remains a broader cultural interest in psychedelics beyond psychotherapeutic application. Reference to psychedelics can be found in art, music, film, and popular writing, and the use of psychedelics spans topics such as creativity, personal optimization, spirituality, recreation, wellness, healing, and social change (Aday et al., 2020). Spiritual hunger (Mahr and Sweigart, 2020), and we would add existential distress associated with climate change and socioeconomic inequality, may also contribute to this broad resumption of interest in psychedelics.

With regard to integration, Western culture is still very much influenced by Cartesian dualism, which creates binaries that polarize and compartmentalize our thinking, for example, mind and body, self and other, or person and nature (Barker and Iantaffi, 2019). We have to wonder if the creation of Western psychology as a science distinct from medicine, social context, and spirituality would be necessary or possible without first cleaving mind from body and person from environment. Abraham Maslow (1968) wrote about the innate drive to self-actualize and return to an essential human core that he saw as obstructed by his (Western) culture. He stated that what socially defines a person as normal is, in reality, “psychopathology of the average,” a widespread sickness of inauthenticity, illusion, and fear. Relatedly, Marx (1844), in his Theory of Alienation, described the social alienation of people as a consequence of capitalism, under which workers are directed toward goals and actions that are not their own, and therefore lose their autonomy to create their lives. The result is alienation from their work, from other people, and from their human nature (and one might add from the natural world, as capitalism resulted in a mass migration to cities and away from the sources of the raw materials of production). From these perspectives, Westerners may particularly need support for integration due to first needing to disintegrate limiting mental structures, then orient and adjust toward new, more authentic, and integrated ways of being that may be unknown to them, within the context of a culture that may define them as abnormal for doing so.

Indigenous worldviews and culture, which do not have a history with Cartesian dualism, may provide invaluable wisdom for Westerners to look to in their understanding of psychedelics, holistic living, and healing practices. There is historical context for doing so. Recent anthropological analysis has provided compelling evidence that we have long underestimated the impact of Indigenous worldview, philosophy, and culture on Western thought. Graeber and Wengrow (2021) compiled extensive evidence that Indigenous American critiques of European culture (particularly related to lack of freedom, collectivism, and egalitarianism) were read widely in Europe in the 1600’s and had substantial influence on the European Enlightenment. Today, interest in Indigenous knowledge and practices is again swelling, likely at least partly driven by the so-called Psychedelic Renaissance.

In this manuscript we seek to review definitions of integration, integration practices, and models of integration. In doing so, we were mindful of the origin of each of these and the degree to which they reflect Western or Indigenous understandings. In the spirit of integration, we seek to synthesize various perspectives and practices.

## Methods

Initial references for this article were identified utilizing PubMed and PsychInfo searches for “psychedelic therapy” and “psychedelic integration” through August 10, 2021. Because our manuscript is not strictly a review of peer-reviewed research but rather of understandings of the concept and related practices, we also searched for these topics on several websites that focus on publishing articles and blog-style posts related to psychedelics, which included Chacruna.net, psychedelicstoday.com, psychedelic.support, and psymposia.com. Our goal was to be extensive in our search for relevant writings, so we also reviewed the reference lists of our initial sources to identify additional references. Our primary sources for integration models include peer reviewed articles and books (including handbooks), for which the quality of peer review was not always evident. We excluded references that did not contain a definition of integration or specific integration practices. We considered an integration model to be present if the authors described an organizational structure for integration activities, a theoretical approach to the process of integration, or a model for navigating the integration process. For references that did not include models, we retained them in a table of integration activities to identify the relative frequency of different types of integration activities mentioned in the literature. This table included thirty-four references. However, we only included a citation of these references if they recommended at least one unique integration activity not already captured in the more comprehensive references. We did not have space, nor was it our intent, to repeat the detailed descriptions of specific integration activities covered at length in some of our references (especially Coder, 2017; Bourzat and Hunter, 2019; Buller and Moore, 2019; Westrum and Dufrechou, 2019; Ortigo, 2021).

Because of the recency of much of the in-depth writing on the concept of integration, we utilized Rodgers (1989) evolutionary concept analysis, which is described as imposing less constraint on the concept being examined than alternative approaches to concept analysis (Pignatiello et al., 2020). This approach involves reviewing the literature for definitions of the concept, characteristics or attributes of the concept, and examples of the concept. This approach corresponded well with our desire to explore and better understand definitions, practices, and models of integration.

## Review of integration definitions, models, and practices

In reviewing the literature, it is possible to identify various degrees of attention to the concept of psychedelic integration. At the broadest level, authors reference the importance of integration without providing many detailed examples or guidance, sometimes not even providing a working definition. At the next level of detail, authors provide detailed examples or recommendations for specific practices that they associate with integration, still often without much guidance or explanation of the purpose of integration. A small number of authors have gone further and elaborated models of integration to provide a detailed framework for an extended integration process. We begin by examining and synthesizing definitions of the concept of integration, followed by a comparison of models of integration, and then review additional integration practices and recommendations within the context of these models.

### Definitions of integration

Many definitions of integration have been put forth. We reviewed published definitions in research articles, books, and various online forums to identify a range of descriptions of the concept and to identify common elements. We also sought to capture the less universal components of the definition that appear to influence integration models and practices.

We identified and reviewed 24 distinct definitions of psychedelic integration (i.e., references that provided their own definition rather than citing another definition). In the great majority of definitions, we encountered the idea of the participant implementing and incorporating the key insights and awareness gained in the psychedelic experience into their life. “Psychedelic integration is a process in which the patient integrates the insights of their experience into their life” (Gorman et al., 2021, p. 8). Most of the definitions also emphasized the need to revisit, work through, and make sense of the material and content of psychedelic experiences. “The term…[refers] to different aspects of a process that includes making sense out of the experience, filtering the content, assimilating and accommodating the experience psychologically, and implementing insights into lasting changes” (Loizaga-Velder and Pazzi, 2014, p. 148). Most authors did not describe the content in their definitions, beyond referring to it as “experience,” though the ones who did elaborate used a range of descriptors, such as “unconscious” or “psychospiritual” content. There was an acknowledgment that content that emerged from psychedelic experiences could be directly beneficial, not obviously relevant, or initially challenging, though none of the definitions pathologized the emergence of difficult or confusing content. “[Santo Daime members] are quite proficient in reinterpreting entheogenic experiences so that difficult, excruciating experiences are reframed as healing, revealing and ultimately positive in the grander scheme of things” (Hartogsohn, 2021, p. 13). Some acknowledged that inadequate social/psychological support may lead to an inability to gain insight or work through less obvious or more challenging content. Nearly all the definitions we reviewed described or implied that integration is a process, one that may take significant time and effort, and without which, insights gained are likely to fade without actualizing meaningful change (Richards, 2017). Some of the definitions focused on the near-term dimension and necessity of post-session support, sometimes referred to as aftercare, while others focused on the longer-term process of internalizing change, prolonging and maximizing benefits, and moving toward greater balance and wholeness internally and with the world (Coder, 2017). Finally, many of the definitions implied or stated that one needed to implement, make use of, bring forward, or otherwise engage in practices to integrate their psychedelic experiences into their lives. “Integration is the process of bringing separate elements together into a whole…and anchoring them into our lives” (Bourzat and Hunter, 2019, p. 179).

After reviewing and identifying common elements of many definitions, we propose the following synthesized definition of integration as it relates to psychedelic experiences:

*Integration is a process in which a person revisits and actively engages in making sense of, working through, translating, and processing the content of their psychedelic experience. Through intentional effort and supportive practices, this process allows one to gradually capture and incorporate the emergent lessons and insights into their lives, thus moving toward greater balance and wholeness, both internally (mind, body, and spirit) and externally (lifestyle, social relations, and the natural world)*.

### Models of integration

We were able to identify ten well-elaborated approaches to integration. Notably, all of these have been published since 2017, which reflects the recent shift in attention to the concept of integration. We considered an integration model to be present when the authors provided a theoretical basis or organizational framework for the various integration practices they described. The identified integration models were primarily based on Indigenous worldviews and practices, Transpersonal Psychology, Jungian Psychology, Acceptance and Commitment Therapy, Psychodynamic Psychology, Somatic Psychology, Nature Relatedness, Biopsychosocialspiritual Models, and Harm Reduction. It should be noted that some of these underlying theories were developed with consideration to altered states of consciousness and psychedelic experiences, while others were originally developed for general psychotherapy. Additionally, some of the models are more focused on the mind, while others are more holistic. Next, we review each model of integration and then contrast and synthesize them.

The first model, called Visionary Plant Medicine Integration, is directed specifically toward individuals who have partaken in ceremonial approaches to plant-based psychedelics (Coder, 2017). The author draws from Transpersonal Psychology and cites a number of Indigenous shamanic experiences (including those utilizing mushrooms, ayahuasca, San Pedro, and iboga) to create a holistic approach to integration organized around seven domains of integration practices. These include Reflection, Inner Listening and Creative Expression, Psychospiritual Practice, Meaning Making, Spaciousness and Time, Nature and Grounding, Physical Care, and Cultivating Virtues and Turning Outward (after introspection and self-care). Specific integration practices are presented within each domain, with some guidelines and rationale provided for choosing specific practices.

An integration model called the Holistic Model for a Balanced Life includes domains of Body, Mind, Spirit, Community (including personal relationships), and Natural Environment (Bourzat and Hunter, 2019). The creators of this model organize integration around a three-part framework of returning from the psychedelic experience (with a focus on capturing a narrative of the experience), understanding the experience (identifying themes present within the experience and decoding the content), and implementing concrete integration practices. The model draws from interpretations of Indigenous understandings and practices. Congruent with the Holistic Model for a Balanced Life, the focus is on integrating body-centered, mind-centered, spirit-centered, community-centered, and environment-centered experiences, followed by sharing one’s transformations with the world, for the purpose of moving oneself and one’s life in the direction of holism. Specific examples and integration practices are provided, along with common themes that emerge during and after psychedelic experiences.

Buller and Moore (2019) provide an organizing structure similar to a biopsychosocialspiritual model to organize a large number of potential integration practices, while not explicitly stating an underlying theory. Their Realms of Integration model consists of domains of relationships, mental and intellect, mind-body, environmental, spiritual, and lifestyle (including career). Although the organizational structure is presented, it is not specifically implemented. A comprehensive list of integration practices is presented, though not all are elaborated upon or clearly connected to the integration model.

Westrum and Dufrechou (2019) offer a model that makes reference to many theories of psychotherapy, but which primarily draws from Transpersonal Psychology while incorporating spirituality and ritual. While no overarching organizational model is provided, there is a focus on psychological, spiritual, existential, ritual, social/communal, and somatic aspects of integration. Psychoeducational content, theory, and corresponding structured integration activities are included. There is a framework for one’s approach to integration, which goes by the acronym SAFETY. The letters stand for security (to allow one to move outside comfort zones), accessible (to focus on available and incremental change process), fluidity (being in flow, particularly around obstacles), empowering (moving toward your authentic self), transformational (capturing big transformational experiences), and yours (the personal nature of the experiences and change).

Gandy et al. (2020) utilized nature relatedness and contact with nature as a foundation to explore the congruence and overlap with psychedelics with psychological concepts such as neurobiology, connectedness, mystical/transcendent experiences, and mindfulness. The authors propose to utilize nature and nature-based rituals in psychedelic integration, while also ways to incorporate natural elements into mindfulness training and talk therapy. They do not provide a formal model of integration, but do provide proposals for incorporating nature to broaden treatment options and enhance inner and outer connectedness.

Gorman et al. (2021) developed the Psychedelic Harm Reduction and Integration Model, which the authors describe as being transtheoretical, drawing from mindfulness-based, psychodynamic, psychedelic-assisted, and harm reduction approaches to psychotherapy. Reference is also made to incorporating additional approaches when appropriate, such as person-centered, somatic, motivational interviewing, ACT, or internal family systems. This approach is described not as a treatment modality or a technique, but rather as a model for facilitating psychedelic harm reduction and integration via psychotherapy. Accordingly, rather than focusing primarily on integration practices or activities, the article focuses on clinical issues and challenges that may arise following psychedelic experiences. They provide recommendations for working with clients who have had challenging psychedelic experiences, common fears that arise (e.g., fear of ego dissolution), increased sensitivity, or somatic experiences, while also focusing on maintaining benefits and supporting clients’ unfolding process.

Ortigo (2021) incorporates existential, spiritual, and unconscious aspects of psychedelic experiences, with descriptions of integration practices that address each of these areas. The author incorporates Jungian views of the psyche and personality, along with elements of transpersonal psychology. The author develops a phenomenological model called Modes of Experiencing, focused on experiencing the world through thought, emotion, body, and behavior. These are then situated within a broader Psychedelic Inclusive Model of the Psyche, which expands beyond the conscious aspects of experience to address the integration of psychedelic experiences across conscious and unconscious aspects of the psyche, and in relation to hypothesized collective dimensions of the psyche.

Cohen (2017) focuses on the integration of Ayahuasca ceremonies and investigates participants’ experiences through the Jungian lens in developing a psycho-spiritual framework for integration. This research employs Jungian psychology, particularly Individuation as it pertains to the psyche, to describe the integration process. It proposes that for the ego to embrace transformation, there needs to be harmony between the conscious and unconscious, either through an establishment or reunification of that relationship, a bid for wholeness. This approach categorizes five emerging themes: pre-ceremony life experiences, the ceremony, post-ceremony integration, process and practices during and after integration, and ayahuasca itself, across dimensions of emotional, psychological, somatic, and spiritual processing, and connects it with the role of the conscious and unconscious. Although focused on ayahuasca, this approach appears applicable to other psychedelics as well.

Sloshower et al. (2020) elaborate a manualized protocol for the application of ACT to psilocybin-assisted therapy for depression, including a focus on preparation, support/guiding, and integration. The ACT Hexaflex model is utilized, which corresponds to the six core principles of ACT: present moment contact, acceptance, diffusion, self as context, values, and committed action (Hayes et al., 2011). The authors report that ACT was chosen due to perceived congruence with psychedelic and mystical experiences, though it was also chosen in part due to its efficacy in treating depression, not being primarily focused on symptom reduction, and ability to make use of psychological flexibility. In this model, the initial stage of integration does not incorporate ACT techniques but instead focuses on eliciting the client’s narrative of the experience. The focus then shifts to identifying parallels in the client’s experience to ACT principles, reinforcing examples of psychological flexibility, reflecting on changes that have occurred, discussing values and how the person is living or not living them, discussing cognitive and behavioral patterns theorized to impact depression, behavioral activation to put values into practice, and implementing ongoing mindfulness practice. Integration practices described in the article are largely therapist initiated and correspond to the ACT model.

Watts and Luoma (2020) propose the ACE (Accept, Connect, and Embody) model, informed by the six processes of the psychological flexibility model, which are part of Acceptance and Commitment Therapy. The model provides a framework for clinicians to support patients in their experience of challenges (Accept), move toward positive aspects (Connect), and promotion of embodied action (Embody) necessary to internalize change. Integration in the ACE model involves three stages. In the first stage, the therapist is non-directive, validating the patient and guiding them through unresolved experiences. The second stage is more structured and helps the patient with meaning-making, creating awareness, and translating insights. The third stage is more directive, with the therapist helping the patient come up with goals and identifying behavioral change. While similar to the Sloshower et al. (2020) model, this approach does not incorporate all aspects of ACT.

#### Contrasting models

In comparing and contrasting the above models, it was challenging to compare them all concurrently because they have different organizational structures (i.e., organized according to integration practices, aspects of the person, model of psychotherapy, or a combination of these), different worldviews (Indigenous and/or Western psychological), different scope (e.g., elaborate focus on the mind versus other dimensions of human experience), and different target audiences (i.e., therapists specifically, facilitators broadly, journeyers, or all of the above). However, it became more feasible to make connections and contrasts across models by first comparing the ones primarily based on theories of psychotherapy and targeted toward therapists (i.e., the last four models above) from the ones that are more holistic or biopsychosocialspiritual and directed toward an audience beyond therapists (i.e., the first six models above). All models are represented in Table 1 for direct comparison, though as noted detail is lost, particularly with the psychological models.

**TABLE 1:** Comparison of integration models with resulting synthesized model of integration.

Model (Author)	Domains					
Synthesized Model of Integration	Mind/emotional/contemplative	Bodily/somatic	Spiritual/existential/ritual	Lifestyle/action	Relational/communal	Natural world
Visionary Plant Medicine Integration (Coder, 2017)	Inner listening, reflection, creative	Physical care, time/space	Spiritual practice, meaning making	Cultivating virtues	Turning outward	Nature and grounding
Holistic Model for a Balanced Life (Bourzat and Hunter, 2019)	Mind	Body	Spiritual	Sharing	Community	Nature
Realms of Integration (Buller and Moore, 2019)	Mental/Intellect	Mind-body, surroundings	Spiritual	Lifestyle/career	Relationships	
SAFETY (Westrum and Dufrechou, 2019)	Psychological (Transpersonal)	Somatic	Spiritual/existential/ritual		Social/Communal	
Nature Contact (Gandy et al., 2020)	Psychological (Nature-based)	Affective	Mystical/Awe			Nature relatedness
Psychedelic Harm Reduction and Integration (Gorman et al., 2021)	Psychological (Transtheoretical)	Somatic	Spiritual/mystical	Harm reduction		
Psychedelic Inclusive Model of the Psyche (Ortigo, 2021)	Psychological (Jungian, Transpersonal)	Body	Spiritual/mystical	Behavior		
Psycho-Spiritual Integration Process (Cohen, 2017)	Psychological (Jungian)	Somatic	Psychospiritual			
Acceptance and Commitment Therapy (Sloshower et al., 2020)	Psychological (ACT)			Behavior change		
Psychological Flexibility Model (Watts and Luoma, 2020)	Psychological (ACT)			Behavior change		

Reflecting first on the holistic models of integration practices, several attempt to incorporate Indigenous knowledge and practices. One limitation here, as with our own overview of Indigenous practices, is that to our knowledge (based on author bios), none of the authors identify as Indigenous themselves. These models could also be broadly described as biopsychosocialspiritual models (Saad et al., 2017). As such, they expand beyond internal experience and the psychological/emotional process. Most of these models share a focus on several of the following aspects of experience: relational and community, lifestyle and action, the natural world, spiritual and existential, mind and contemplation, and bodily and somatic experience. These models view people as multifaceted and tend to identify the causes of suffering as rooted in imbalance or lack of integration of these interconnected aspects of existence (Saad et al., 2017). Particularly for the approaches that invoke Indigenous worldviews, the need for integration could be seen not as resulting from psychedelic experiences but as a condition of Western culture, with its tendency to place disproportionate value on mind, materialism, and behavior (i.e., Coder, 2017; Bourzat and Hunter, 2019). As such, integration is about more than processing the psychedelic experience or overcoming specific difficulties, even if these might catalyze the process, but are about bringing balance and alignment to one’s whole existence. It is assumed within each of these models that integration is often a lengthy process or even the core project of our lives.

By contrast, the psychotherapy models tend to delve in-depth into one or two dimensions of the holistic models (particularly the “mind” dimension). However, we do note that one model (Gorman et al., 2021) falls somewhere between by applying a transtheoretical approach that incorporates a range of psychotherapy approaches and a more biopsychosocial model. The fact that these models focus on fewer dimensions is not to say these approaches are not useful, but they clearly have a primary focus on the mind and mental health, whereas the holistic models equally value and seek a balance between all dimensions of human experience and seek to touch on all of them as part of the integration process. The psychotherapy models also tend to view integration as something that is necessitated by psychedelic experiences rather than a more universal need among Westerners. Thus, the therapy models focus more on processing, making use of, and making changes based on the psychedelic experience, whereas the holistic models are more likely to assume that people are drawn to psychedelic experiences in the first place by their imbalances and lack of integration. Still, for psychotherapists not well versed in incorporating somatic, spiritual, natural, and community-focused interventions into their practice, the psychotherapy models are likely to be an accessible entry point to supporting clients who are utilizing psychedelics. A psychotherapeutic framework may “meet them (clients and therapists) where they’re at,” though it may not prepare the client or therapist for experiences that transcend typical psychotherapeutic process and content.

**FIGURE 1:**
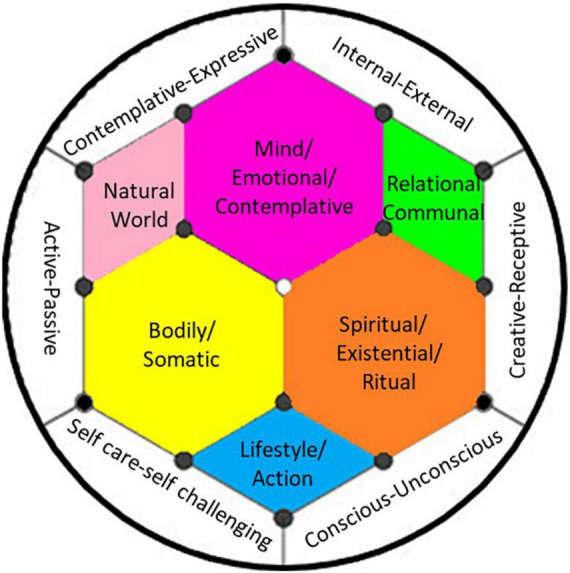
Synthesized model of integration: The hexagon reflects six interconnected domains of existence, with the more personal ones toward the center. The outer ring reflects continuums on which integration activities can be placed. The goal is a balance of integration activities addressing all domains of living.

We concluded our review of integration models by capturing and contrasting the primary components of each model, which resulted in our proposal of a new Synthesized Model of Integration (Figure 1). The six areas in the center of the model are facets of the person and their experience (i.e., mind/cognitive/emotional, bodily/somatic, spiritual/existential, natural world, relational/communal, and lifestyle/action). Our model also includes six continuums on which to organize integration practices along the outer ring of the model. These include the degree to which the practice is more conscious or unconscious, more internally or externally focused, more creative or receptive, more passive or active, more outside of one’s comfort zones or gentle self-care, or more contemplative or expressive. We suggest that journeyers reflect on their preferences on each of these continuums and choose a balance of integration practices that addresses each side of each continuum while also drawing from each domain of experience in the Synthesized Model of Integration. Taken together, a balance of integration practices is congruent with the intention to move toward holism.

### Integration practices and activities

Integration practices and activities provide the most granular level of analysis of the concept of integration. An expansive range of practices have been suggested in the literature, often without much rationale or context. This likely accounts for some of the confusion about what integration is and how to best practice it. On the one hand, many writings about integration provide only a few examples of integration, while others provide extensive lists. This can lead to a lack of direction at the one extreme or a sense of overwhelm and confusion at the other extreme. The integration models and frameworks already discussed provide guidance for choosing and implementing integration practices. Our synthesized model may provide further framework when attempting to resolve gaps within and contradictions between integration models.

While the great majority of writings we identified on the topic of integration were brief and did not correspond to a theory or model, we reviewed a large number of additional writings to identify a comprehensive list of integration practices not included with the models we have focused on thus far. Notably, the vast majority of integration practices we found in briefer writings had already been referenced in relation to one or more of the integration models. We initially hoped to organize these integration practices according to our Synthesized Model of Integration, though many of the integration practices cut across domains (e.g., mind-body integrative practices). Instead, we coded integration activities by theme (see Table 2).

**TABLE 2:** Summary of integration practices by theme.

Theme	Integration practices
Artistic/creative	Drawing Mandalas (multiple) Drawing (multiple) Painting (multiple) Art exercises (multiple) Creative expression (multiple)
Music/singing	Listening to music Playing music (Grof, 2008) Chanting/Singing (multiple) Drumming (Kaufman and McCamy, 2019)
Movement/somatic	Drumming (Kaufman and McCamy, 2019) Yoga (multiple) Dance (multiple) Qigong (Buller and Moore, 2019) Tai Chi (Coder, 2017; Bourzat and Hunter, 2019) Progressive Muscle Relaxation (Westrum and Dufrechou, 2019) Walking in nature (multiple) Active movement: Hiking, Bicycling, Sailing, Martial Arts (Coder, 2017; Bourzat and Hunter, 2019) Exercising (multiple) Massage (multiple) Acupuncture (Buller and Moore, 2019) Bath soaks/Shower (Bourzat and Hunter, 2019; Westrum and Dufrechou, 2019) Essential oils/Aromatherapy (Bourzat and Hunter, 2019; Buller and Moore, 2019) Sweat/Sauna (Bourzat and Hunter, 2019) Sensory deprivation/Float Tank (Bourzat and Hunter, 2019) Laugh (Buller and Moore, 2019) Sexual life/needs (Buller and Moore, 2019)
Diet/health practices	Healthy diet (multiple) Fasting (Westrum and Dufrechou, 2019) Naturopathy (Frecska et al., 2016) Colonic irrigation (Frecska et al., 2016)
Quiet time/downtime	Self-reflection/Introspection (multiple) Rest (multiple) Reading (multiple) Time (multiple) Space for emotions (Buller and Moore, 2019) New hobbies (Ortigo, 2021)
Journaling	Journaling (multiple) Dream Journal (Ortigo, 2021)
Therapy/mind focus	Therapy Bibliotherapy (Frecska et al., 2016) Psychotherapy (multiple) Family Constellation therapy (Labate and Cavnar, 2014) Internal Family Systems (Morgan, 2020) Sculpting (Grof, 2008; Labate and Cavnar, 2014) ACT Model (Sloshower et al., 2020; Gorman et al., 2021) Group therapy (Trope et al., 2019) Mindfulness (Gorman et al., 2021) Harm Reduction (Gorman et al., 2017, Gorman et al., 2021) Transpersonal/Psychodynamic (Passie, 2009) Values Clarification/Belief work/Virtues (multiple) Assimilating memories (Richards, 2015; Belser et al., 2017) Emotions work (Kaufman and McCamy, 2019) Reinforcing new habits (Bourzat and Hunter, 2019) Insights/Values into action (multiple)
Meditation/mindfulness	Mindfulness practice (multiple) Meditation (multiple) Walking meditation (Westrum and Dufrechou, 2019) Shinrin-Yoku practice (Gandy et al., 2020) Body scan (Westrum and Dufrechou, 2019) Breathing techniques (multiple) Breathwork (Buller and Moore, 2019; Westrum and Dufrechou, 2019) Mundane activities done with mindfulness (Westrum and Dufrechou, 2019; Bast, 2020)
Nature	Time in nature (multiple) Horticulture (Gandy et al., 2020) Nature walk (multiple) Talk therapy in nature (Gandy et al., 2020) Shinrin-Yoku practice (Gandy et al., 2020) Favorite places in nature (Bourzat and Hunter, 2019
Creating space/ritual	Creating an altar/Sanctuary (multiple) Physical comforts (Bourzat and Hunter, 2019) Arrange comfortable workspace (Buller and Moore, 2019) Organizing/Cleaning (Bourzat and Hunter, 2019) Nourishing environment (Fadiman, 2011)
Spiritual/existential	Spixritual practice (multiple) Intention setting (Frecska et al., 2016; Kettner et al., 2021) Mantra work (Coder, 2017) Gratitude practice (Coder, 2017) Prayer (multiple) Tarot/Medicine (Westrum and Dufrechou, 2019) Sage/Smudging (Buller and Moore, 2019) Self-Awareness/Individuation practice (Ortigo, 2021) Astrology (Buller and Moore, 2019) Inner listening (Coder, 2017) Connect with spiritual mentor/community (Buller and Moore, 2019) Practice openness, presence, awareness (Coder, 2017) Exploring relationship with death (Westrum and Dufrechou, 2019) Reflect on elementals (Coder, 2017)
Dreamwork/Symbolic interpretation	Dream work (multiple) Shadow work (Westrum and Dufrechou, 2019) Dream journaling (Ortigo, 2021) Interpreting symbols (multiple) Exploring metaphors in nature (Coder, 2017)
Community/activism	Community Participation and Support (multiple) Volunteer (Buller and Moore, 2019) Activism (Bourzat and Hunter, 2019) Service (Bast, 2020) Donating (Bourzat and Hunter, 2019) Serving in hospice care (Bourzat and Hunter, 2019) Bring beauty in the world (Coder, 2017) Practice love toward world (Coder, 2017)
Relational/interpersonal	Boundary setting (Bourzat and Hunter, 2019; Buller and Moore, 2019) Building connections (Buller and Moore, 2019) Writing letter to loved one (Bourzat and Hunter, 2019) Reaching out for help (Bourzat and Hunter, 2019) Non-sexual touch/physical closeness (Passie, 2009) Interpersonal closeness (Passie, 2009) Practice love toward others (Coder, 2017) Sharing circles/groups (multiple) Time with loved ones and children (Buller and Moore, 2019)

While some themes correspond directly to the facets of our synthesized model (e.g., nature, spiritual/ritual), others were further broken down into subthemes (e.g., bodily/somatic largely corresponds to the diet/health and somatic/movement themes). We note that some integration practices in each theme bridge across multiple facets of our model. For example, outdoor activities like hiking incorporate both nature and physical activity. This may be desirable if the goal is to achieve balance and integration across aspects of living, even if it makes categorization of activities less clear. We are reminded that while categories help us to make sense of the world, they ultimately are mental constructions, and that such categorical thinking reflects a Western worldview (Limb and Hodge, 2008). Lastly, we note that some of our themes involve practices that are supportive of the overall project of integration, though do not correspond to facets of our synthesized model, such as downtime/quiet time or creating space/setting for integration. These are not aspects of individuals but are part of one’s set and setting that can support integration.

### Expectations and approach to integration

Beyond models and practices, our primary sources emphasize important expectations (part of one’s set) that can impact how one approaches integration. First, our primary sources all emphasize that integration requires active effort to revisit and work with psychedelic experiences and content that emerges from them. Without such active effort, valuable lessons tend to fade, and difficult experiences can reinforce traumas or existing patterns and defenses. Contrary to common belief, rather than doing the healing for us, psychedelics may give us an experience of and orientation toward wholeness, along with insight into the barriers and misalignments that will need to be addressed to continue toward or maintain wholeness (e.g., Coder, 2017).

Second, most of our primary references describe integration as not just an event or brief phase but a long-term process. While some changes may occur quickly and permanently, and the initial integration of a fresh experience is particularly important, many aspects of psychedelic experiences may continue to unfold gradually or even over the course of one’s lifetime as they become relevant and take on new meaning during different phases of one’s life (e.g., Coder, 2017). Relatedly, there was a theme of integration requiring time and space, or creation of supportive settings (including physical spaces such as altars or meditation sitting areas). Without regular practice and lifestyle changes that allow time to reflect, engage in intentional integration practices, and engage with others, one is unlikely to have an adequate container in which to hold and work with the entirety of their psychedelic experiences.

Some of our references suggested that integration may be more accessible when incremental (e.g., Westrum and Dufrechou, 2019), while also acknowledging that important changes may be more accessible when closer in proximity to one’s psychedelic experiences (e.g., Bourzat and Hunter, 2019). Paradoxically, the bigger and more transformational the experience, the more incremental the approach to integration may need to be, as they can take time to digest, and any changes required may take time to implement. For more profound psychedelic experiences, like any other profound experience in life, it would make sense to continue to reflect on the experience and may new interpretations and value from it over the lifetime.

The integration process may also be more congruent with the psychedelic experience if the journeyer can allow it to unfold organically, rather than pushing for an overly task-oriented approach to integration practices (Bourzat and Hunter, 2019). In this sense, integration models can help one to be aware of the many areas of existence and how integration practices can nourish them, but not applied so rigidly as to ignore one’s intuition of what is needed but should not be used so rigidly as to ignore what intuitively feels like what is needed. The concept of “inner healing intelligence,” is often cited to refer to the innate knowledge and tendency to orient toward healing, growth, and wholeness (Grof, 1996).

Although most of the models encourage starting with contemplation, they do not encourage isolation. Integration is often described as ideally including supportive communities and supportive others (Coder, 2017). It may also include a range of professionals, such as therapists, dieticians, acupuncturists, meditation teachers, massage therapists, spiritual teachers, etc. (Bourzat and Hunter, 2019). The presence of other people as part of the integration process can help us overcome the myth that we are self-contained individuals, with interpreting and capturing insights from psychedelic experiences, and by providing interpersonal grounding or regulation of emotion. However, there generally is a caution given about including those who do not understand or support psychedelic experiences or the sensitive state one is in afterward (Coder, 2017).

Finally, bringing forth one’s insights, values, changes, and gifts to the world are treated as a later step in the process of integration (e.g. Coder, 2017; Bourzat and Hunter, 2019; Sloshower et al., 2020). This appears partly practical in that one needs to reflect on what they would want to share with the world and why, but can also be a strategic buffering of potential inflationary experiences where one feels compelled to evangelize or make impulsive changes based on their experiences without first adequately exploring and internalizing them. Not all the models addressed action in the world as part of integration, though we noted that the models addressing action did not take an overly linear view that one must change oneself before changing the world, when in fact these can be mutually reinforcing processes. We are reminded of pioneering action researcher Kurt Lewin, and his circular process of observing, reflecting, planning, acting, and then repeating (Glassman et al., 2013).

## Discussion

The concept of psychedelic integration has been criticized as lacking clear definition, having no historical context, and containing a confusing range of activities (e.g., Sloshower et al., 2020). It appears this critique initially stemmed from the historical lack of theories and models to conceptualize the integration process, but more recently from a slew of independently developed definitions and approaches to integration. While psychedelic researchers and practitioners have long highlighted the importance of integration (e.g., Grof, 1980), little structure was provided until recently. Accordingly, we focused our attention on writings that included models of integration, two of which were published in 2017 and the remaining seven in 2019 or later, reflecting the very recent expansion of scholarship on the topic. While it is beyond the scope of this article, we believe that integration does have a historical context and can broadly be seen as a therapeutic common factor, that is a process inherent to, if unnamed by, most theories of psychotherapy (Wampold, 2015). The assumption that insights need support and ongoing processing to result in lasting change is apparent in most theories of psychotherapy, such as through the use of homework or the incorporation of relapse prevention or behavioral activation.

We were particularly aware of the increasing popularity of ACT in relation to psychedelic-assisted therapy, and it may prove to be a very useful framework, though to our knowledge ACT was not developed with psychedelics in mind and therefore may result in fitting client experiences to the theory rather than fitting the theory to the broad range of experiences that come out of psychedelics. While ACT and other psychotherapy-based integration models may provide structure to work on the psychological and behavioral aspects of psychedelic experiences (e.g., meaning making, changing thinking or emotional patterns, or working through traumas), they do not easily incorporate a holistic model of living, possibly with the exception of transtheoretical models that incorporate a range of additional theories to focus on aspects of experience beyond the mind and behavior (i.e., Gorman et al., 2021). Research has shown that Westerners are likely to focus primarily, but not exclusively, on psychological or mental health-related intentions even when they participate in facilitated shamanic or neoshamanic ayahuasca experiences (Bathje et al., 2021). However, the same study also found that participants experienced a much broader range of impacts from their participation than they intended, more reflective of holistic worldviews. These included transcendent experiences, ego death, feelings of unity or interconnectedness, greater engagement in activism, decreased attachment to materialism, powerful somatic/energetic experiences, mind-body integrative experiences, spiritual/mystical experiences, changes in spiritual beliefs, dietary changes, improvements in physical ailments, and emergent experiences that might be misunderstood as mania or psychosis. While psychotherapeutic models may be particularly helpful for assisting journeyers who have psychotherapeutic intentions or significant mental health needs, these models may not provide a broad enough framework for practitioners or journeyers to respond to this full range of psychedelic experiences. We found that the integration models that were influenced by biopsychosocialspiritual and/or Indigenous perspectives addressed a broader range of themes that may arise in psychedelic experiences, and therefore appear indispensable to psychedelic integration.

We also noted recent critique of the tendency among psychedelic researchers and practitioners to impose spiritual interpretations of psychedelic experiences (e.g., Sanders and Zijlmans, 2021). While we agree that imposing belief and meaning is problematic and we support the use of neutral language or use of the participant’s own language, it would be similarly problematic for practitioners not to broach the topic of religion and spirituality or fail to ask participants if they would like to incorporate such focus into their work. We believe integration will be most complete when expanding to address the full range of experiences people have with psychedelic substances. In fact, if we utilize more holistic definitions of integration that value balance, then overly mentalizing the psychedelic experience may create further imbalance by reinforcing our already powerful cultural preference for individualism, behaviorism, and cognition without adequately attending to the spiritual/existential, communal/relational, or the natural world (Limb and Hodge, 2008). In attempting to work holistically, those assisting journeyers with integration will need the humility to recognize the limitations of their training and knowledge, and limitations of their cultural conditioning and worldview. A skilled range of collaborators and referral sources are likely to be valuable in facilitating the integration process.

In examining the existing models of integration, we were able to capture a comprehensive list of integration practices (see Table 2), along with a rationale for their breadth. We note that the cultural sources of these integration practices are not always cited, but that many of them come from non-Western religious, cultural, or healing practices (e.g., yoga, use of sage, qi gong, sweat lodges, mindfulness meditation, etc.). In contrasting the models, we were able to contrast existing integration models (see Table 1) and incorporate them into a Synthesized Model of Integration that further captures aspects of each (see Figure 1). We suggest using this model to develop a balanced approach to integration that addresses each of the six facets of experience and reflect on each side of the six continuums on the outer ring of the model to be sure to choose a balance of integration activities. For those seeking more structure and guidance regarding specific integration practices and how to implement them, journeyers may consult an integration professional and review one of the integration handbooks reviewed in this article for ideas (e.g., creating an altar, getting in touch with the body via somatic practices, reflecting on values, or dreamwork). It seems ideal to have a relationship established with a professional prior to psychedelic experiences so they can aid in preparation and be available in the event of a challenging experience where immediate support is needed. However, we note with humility that our model and its application will benefit from continued refinement, elaboration, and research.

Lastly, it is important to highlight the context, or set and setting, for optimal integration. Because there are so many options for integration, we suggest beginning to address integration during the preparation stage, prior to psychedelic experiences. This can include taking an inventory of one’s resources and abilities that will support integration (e.g., artistic skills, wellness practices, favorite places in nature), reviewing integration resources such as the models referenced in this article, reflecting on one’s current degree of satisfaction and sense of balance among the six facets of living in the Synthesized Model of Integration, planning to put active effort into integration, planning time and space for integration, and asking an integration professional or supportive people to be available for sharing one’s experience. After psychedelic experiences, integration can be supported by reviewing one’s original intentions for the psychedelic experience, determining which integration practices are most relevant, committing to regular integration practices, identifying relationships and communities that can support integration, carving out time for integration, and creating or finding physical spaces at home, public spaces, or in nature that support the ongoing unfolding of psychedelic experiences over time. Without time and space, one may end up taking the path of least resistance by attempting to integrate the psychedelic experience into their ordinary state of consciousness and life structure rather than being changed by the experience. This process might also be observed at the societal level, as Western culture attempts to constrain and assimilate psychedelics into existing structures, such as capitalism or the medical model. One’s resources, responsibilities, and privileges are likely to come into play with regard to the opportunity to more fully engage the integration process and implement life changes. For individuals with more constraints and fewer resources, an incremental and gradual approach may be particularly important for integration, while highly resourced and privileged individuals may need to be encouraged to slow down and reflect on change and action.

In conclusion, much work has been done to elaborate on the topic of integration in the past few years. New models and extensive guides for integration practice have been developed. Because of the recency of these efforts, ours is the first article to synthesize definitions, models, and practices related to integration. We hope this will bring more intentional focus to researching and further developing this crucial stage of the psychedelic experience.

## Author contributions

GB contributed to all sections and analysis. EM contributed to literature search, introduction, results, analysis, and manuscript formatting. MK contributed to literature search, introduction, results, and analysis. All authors contributed to the article and approved the submitted version.
